# Leveraging explainability for understanding object descriptions in ambiguous 3D environments

**DOI:** 10.3389/frobt.2022.937772

**Published:** 2023-01-04

**Authors:** Fethiye Irmak Doğan, Gaspar I. Melsión, Iolanda Leite

**Affiliations:** Division of Robotics, Perception and Learning, School of Electrical Engineering and Computer Science, KTH Royal Institute of Technology, Stockholm, Sweden

**Keywords:** explainability, resolving ambiguities, depth, referring expression comprehension (REC), real-world environments

## Abstract

For effective human-robot collaboration, it is crucial for robots to understand requests from users perceiving the three-dimensional space and ask reasonable follow-up questions when there are ambiguities. While comprehending the users’ object descriptions in the requests, existing studies have focused on this challenge for limited object categories that can be detected or localized with existing object detection and localization modules. Further, they have mostly focused on comprehending the object descriptions using flat RGB images without considering the depth dimension. On the other hand, in the wild, it is impossible to limit the object categories that can be encountered during the interaction, and 3-dimensional space perception that includes depth information is fundamental in successful task completion. To understand described objects and resolve ambiguities in the wild, for the first time, we suggest a method leveraging explainability. Our method focuses on the active areas of an RGB scene to find the described objects without putting the previous constraints on object categories and natural language instructions. We further improve our method to identify the described objects considering depth dimension. We evaluate our method in varied real-world images and observe that the regions suggested by our method can help resolve ambiguities. When we compare our method with a state-of-the-art baseline, we show that our method performs better in scenes with ambiguous objects which cannot be recognized by existing object detectors. We also show that using depth features significantly improves performance in scenes where depth data is critical to disambiguate the objects and across our evaluation dataset that contains objects that can be specified with and without the depth dimension.

## 1 Introduction

**FIGURE 1:**
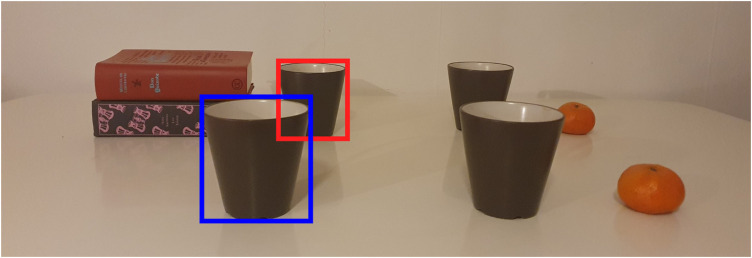
An example illustrating the motivation behind using depth to improve referring expression comprehension. In this example, when the user’s object description is ‘the mug next to the books’, the robot can suggest the mug in the blue bounding box in RGB or the one in the red bounding box in RGB-D. Best viewed in color.

When humans and robots work on tasks as teammates, it is critical for robots to understand their human partners’ natural language requests to successfully complete the task. During the task, the robot can encounter many challenges. For instance, when the robot is asked by its human partner to pick up an object, there can be misunderstandings caused by failures of speech recognition or the use of object descriptions that are unknown to the robot. Another challenge can be ambiguous requests (e.g., the human partner’s object description might fit more than one object). In these cases, the robot should be able to make reasonable suggestions to its partner by using the familiar concepts in the request. For example, it should suggest the objects that fit the description instead of just saying it couldn’t understand the request. While handling these challenges, the depth dimension also plays an important role for the robot. For instance, consider a robot located in the environment of Figure 1, helping a user pick up a described object. In this scenario, if a user asks the robot to pick up ‘the mug next to the books,’ it can aim to take the incorrect mug (i.e., the one in the blue bounding box) using the RGB scene because this mug is the closest to the books in 2D. Alternatively, if the robot can obtain the RGB-D scene and use the depth dimension to solve the problem, it can aim to take the correct mug (i.e., the one in the red bounding box), which is the closest to the books in 3D. Therefore, the depth dimension is critical in this scenario to understand the user’s object descriptions.

People can identify objects with the help of referring expressions, which are phrases that describe the objects with their distinguishing features. In robotics, comprehending object descriptions has been studied extensively. Prior work has focused on situated dialogue systems (Kruijff et al., 2007; Zender et al., 2009), probabilistic graph models (Paul et al., 2016), and learning semantic maps (Kollar et al., 2013). Recent work on comprehending referring expressions has also employed models based on deep learning (Hatori et al., 2018; Shridhar and Hsu, 2018; Magassouba et al., 2019; Shridhar et al., 2020).

In this paper, we propose a method to comprehend users’ expressions using deep neural networks’ explainability in real-world, ambiguous environments. Although recent human-robot interaction (HRI) studies evaluate the importance of explainable AI for different tasks (Siau and Wang, 2018; Edmonds et al., 2019; Sridharan and Meadows, 2019; Tabrez and Hayes, 2019), to our knowledge, this is the first work using explainability to comprehend the user descriptions. Recent models on comprehension of user expressions demonstrate promising results, but they assume the target candidates in a scene are given (Magassouba et al., 2019), or these candidates can be obtained from the existing object detection (Hatori et al., 2018) or localization methods (Shridhar and Hsu, 2018; Shridhar et al., 2020). However, when robots are deployed in the real world, the encountered objects are not limited to the ones that can be detected by the state-of-art object detection or localization models, and it is not feasible to expand these models to localize every object category in a supervised fashion. Even when dealing with detectable object categories, due to environmental conditions such as poor illumination or cluttered scenes, these objects might not be possible to classify. In that case, when the described objects cannot be detected or localized, the existing solutions do not even consider these objects as target candidates. On the other hand, for a more general solution, our approach finds active areas of a scene using the explainability activations of an image captioning module, which is not trained on object-wise supervised fashion and learns a higher-level feature space. Therefore, our method does not require any detectable target candidates to suggest the described regions. This allows our system to handle various objects (including uncommon ones that may not be proposed by existing object detection or localization models) without putting any constraints on object categories or users’ expressions.

In addition to focusing on limited object categories while comprehending referring expressions, most techniques in computer vision and robotics studies have relied on flat RGB images without using the depth dimension (Hatori et al., 2018; Yu et al., 2018; Magassouba et al., 2019). However, depth information plays a critical role in real-world environments, and it was recently shown that depth features could facilitate the comprehension of referring expressions (Mauceri et al., 2019). Consequently, there have been recent attempts to address this challenge using the three-dimensional feature space (i.e., 3D point clouds) (Achlioptas et al., 2020; Chen et al., 2020). Although these studies have shown promising results, in contrast to our system, they have still required candidate objects and selected the target object among the 3D object proposals. To our knowledge, our method is the first one to use explainability in RGB-D images to identify the described object regions in 3D environments.

**FIGURE 2:**
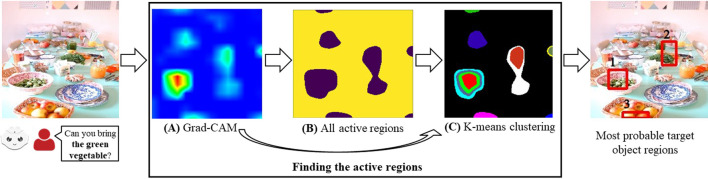
Overview of the Grad-CAM RGB method to find the described object regions for a given RGB scene and a referring expression (the bold part of the user’s expression corresponds to the referring expression). The heatmap generated by Grad-CAM in **(A)**, all active regions in **(B)**, and the results from K-means clustering in **(C)**.

In this work, we first find the active areas of an RGB scene using the explainability module (i.e., Grad-CAM (Selvaraju et al., 2017)), and then we use an unsupervised clustering technique (i.e., K-means) to find the active clusters. These active clusters are proposed as the regions that the robot needs to direct its attention to (**Grad-CAM RGB method** shown in Figure 2). Next, we extend this approach by providing the depth features in the input space and generating the RGB and depth activation heatmaps from Grad-CAM. Then, we obtain the combined activations showing the areas that are active in both of these heatmaps and cluster the combined activations (**Grad-CAM RGB-D method** shown in Figure 5). Our results show that the regions suggested by the Grad-CAM RGB method can be useful for resolving ambiguities. Moreover, compared to a state-of-the-art referring expression comprehension model (i.e., MAttNet (Yu et al., 2018)), the Grad-CAM RGB method performs better in the scenes where several objects match the same description (e.g., multiple similar fishes) and where there are uncommon objects typically not recognized by off-the-shelf object detectors (e.g., an artichoke). Finally, we show that depth features employed by the Grad-CAM RGB-D method further enhance the performance in the scenes where the object descriptions are dependent on the depth dimension and in the evaluation dataset containing depth-dependent and independent features.

### 1.1 Contributions

 Our contributions in this work can be summarized as follows:

• We propose using the explainability of image captioning to improve the effectiveness of referring expression comprehension (**Grad-CAM RGB Method**). To our knowledge, this is the first work employing explainability for comprehending user expressions to direct robots to described objects in the wild, without any restrictions such as detectable or localizable objects.
• We extend Grad-CAM RGB Method to take the depth dimension as an input, and we identify the target object regions from RGB-D images (**Grad-CAM RGB-D Method**). To our knowledge, our work is the first one comprehending referring expressions considering the depth of the objects using explainability.
• We examine the regions suggested by the Grad-CAM RGB method to determine whether these regions can be used for asking for clarification to resolve ambiguities.
• We compare the Grad-CAM RGB method with a state-of-the-art baseline in varied real-world images and show that our method performs better in challenging environments (i.e., scenes with uncommon and similar objects), which robots will more likely encounter in the real world.
• We show that using the depth dimension in the Grad-CAM RGB-D method improves the performance in scenes where the target objects are described with the spatial relations dependent on the depth features and in the whole evaluation dataset, which contains object features both dependent and independent of the depth dimension.

## 2 Background

### 2.1 Referring expression comprehension

In human-robot collaborative settings, referring expression comprehension (REC) is crucial, where the physical nature of the interaction allows humans to expand their natural language expressions with visual cues. Recent advances have taken advantage of this characteristic to improve the comprehension of referring expressions from humans (Mao et al., 2016; Yu et al., 2018; Magassouba et al., 2019; Kamath et al., 2021). In addition, it is common that the expressions given by humans are ambiguous and difficult to interpret by the robot, where interactive methods for the robot to clarify them have been shown to improve the success rate of comprehension (Hatori et al., 2018; Shridhar and Hsu, 2018). However, these methods are usually limited to the pre-learned object categories of the vision algorithm. The work from Shridhar et al. (2020) avoided the use of predefined object categories, but it was still restricted to the target candidates obtained from the DenseCap object localization module (Johnson et al., 2016). Hence, we present a novel approach employing explainability to solve the task of comprehending referring expressions that removes the dependency on using an object detection module that limits the results to the learned object categories.

#### 2.1.1 Spatial referring expressions

It is common that referring expressions contain relational concepts between multiple entities in the scene, and its exploitation has been shown to improve the capability of the models to comprehend those expressions (Zender et al., 2009; Nagaraja et al., 2016; Hu et al., 2017; Shridhar et al., 2020). In particular, these relationships tend to be spatial relations from the point of reference of the user and the robot must be able to cope with this kind of descriptions in order to resolve any ambiguities there might be to eventually identify the right entity in the scene (Ding et al., 2021; Venkatesh et al., 2021; Roh et al., 2022). Ding et al. (2021) present a transformer-based architecture combining the language features with a vision-guided attention framework to model the global context in a multi-modal fashion. Nagaraja et al. (2016) provided CNN features to LSTMs to model spatial relationships between a region and its context regions. Shridhar et al. (2020) proposes a two-stage approach, first generating descriptions of the candidate objects and then finding the best match with the object in the expression.

#### 2.1.2 Using depth for REC

While identifying the spatial relationships among objects, depth information has been shown to improve the task performance (Birmingham et al., 2018). Consequently, studies on referring expression comprehension have also focused on resolving this problem in three-dimensional feature space (Yuan et al., 2021; Thomason et al., 2022). For instance, 3D Point Clouds were used as an input to select the target objects among the detected object candidates (Chen et al., 2020) or segmented 3D instances (Achlioptas et al., 2020). Further, Mauceri et al. (2019) proposed an RGB-D dataset with referring expressions and evaluated this dataset with proof-of-concept experiments. In their experiments, they modified the referring expression generation model of Mao et al. (2016) to take the depth dimension as an input in addition to RGB features. They also used this generation method for comprehension by maximizing the probability of generating the input expression for candidate bounding boxes. Their findings showed pioneering results for our work: additional depth features enhanced the model’s performance. However, their method assumed that the candidate bounding boxes were given or could be obtained by object box proposal systems, but our method does not require any candidate proposals thanks to leveraging explainability of image captioning activations.

### 2.2 Explainability

Explainability has been claimed to offer a viable solution to make intelligent systems more fair and accountable (Barredo Arrieta et al., 2020). There have been several techniques presented in the academia to make machine learning models to be more interpretable (Guidotti et al., 2018), and they have been curated to the variety of existing models, e.g., classifiers (Ribeiro et al., 2016), image captioning (Selvaraju et al., 2017), natural language processing (Alonso et al., 2021), and reinforcement learning (Madumal et al., 2020). There are multiple uses that explainable systems may have, depending on the step of the development and deployment cycle to which the explainability is being leveraged. For instance, explanations may be useful for developers in order to debug their models and be able to understand better their functioning to correct them in the best way possible (Kulesza et al., 2015), for field experts using AI-based systems e.g. to aid in medical diagnosis (Watson et al., 2019), or as integrated part to improve a system’s performance (Hendricks et al., 2018), but also it is crucial for consumers of the technology to understand how the systems work e.g. in bank loan applications and the ‘right to explanation’ from the latest data privacy standards (Adadi and Berrada, 2018).

The broad audience of the field has caught the attention of researchers from a variety of areas that raised concern about the viability of the current explainability solutions to be usable for the general public (Abdul et al., 2018; Wang et al., 2019), stating a clear mismatch between the technical advances and the appropriate practices in the way explanations are presented to the users (Miller et al., 2017). Miller (2019) established specific lines of investigation based on research from the social sciences on explanation that could help make Explainable AI (XAI) systems to be more human-centered, and several works have used it as foundation to study explainability in applications closer to the end-user with an effort to understand their preferences (Ehsan and Riedl, 2020; Liao et al., 2020). In this work, we present a novel use of explainability that enables the robot to act in a more human-centered way when recognizing users’ expressions of its surroundings.

#### 2.2.1 Explainability in human-robot interaction

The embodiment and social factors of HRI add a new dimension to the importance of designing and using explainability with a human-centered approach for robotic applications (Han et al., 2021). The physically embodied nature of robots give them the capacity to expand the interaction to different levels, by using social cues and multiple modalities to convey their explanations. Although there have been advances in explainability techniques that make use of multimodal explanations combining visual approaches with text (Park et al., 2018), currently the majority of explainable embodied agents do not take advantage of it, and most researchers opt for using only lexical utterances for the robot to deliver its explanations (Wallkötter et al., 2021).

Explainability has been shown to be an important tool to use in human-robot collaboration settings. For instance, during an interactive robot learning scenario, explainability may help the human teacher to make better decisions based on the robot’s explanations (Chao et al., 2010; Edmonds et al., 2019), and increase the predictability of the robot’s actions to facilitate collaboration between humans and robots on a shared task (Tabrez and Hayes, 2019). Other examples in the HRI community use explainability for non-experts to understand the causes of unexpected failures in robotic systems (Das et al., 2021). We want to contribute to this body of work by leveraging explainability in specific robotic applications.

#### 2.2.2 Using explainability for advancing the System’s functioning

Explainability has also been used for advancing the systems’ functioning (Ross et al., 2017; Hendricks et al., 2018; Li et al., 2018; Selvaraju et al., 2019). Recent computer vision studies have demonstrated the potential of interpretability to expand the use of explainability beyond the original concept of transparency by using explanations to improve models’ intrinsic functioning. For instance, Selvaraju et al. (2019) aligned the visual explanations obtained from Grad-CAM (Selvaraju et al., 2017) with the human attention heatmaps to improve task accuracy in image captioning and visual question answering tasks. Hendricks et al. (2018) used a similar approach to force a captioning model to generate gender-specific words based on the person region of the image instead of the biased reasons given by gender-stereotyped datasets. Similarly, Ross et al. (2017) improved model generalization by constraining explanations with input gradient penalties.

In other domains, human attention maps have been aligned with the explanations provided by Grad-CAM to improve visual grounding in vision and language tasks (Selvaraju et al., 2019). Further, Li et al. (2018) presented a method to generate more accurate explanations (i.e., attention maps) through supervision in an end-to-end fashion while training the network. In line with enhancing the intrinsic functioning, our work leverages explainability to improve human-robot collaboration, using Grad-CAM (Selvaraju et al., 2017) saliency maps to direct the robot’s attention to the appropriate regions described by the user.

## 3 Proposed method

In this section, we present our method finding the described regions from RGB scenes (Grad-CAM RGB method), and also how we extend this approach to also consider the depth features (Grad-CAM RGB-D method).

### 3.1 Grad-CAM RGB method

For a given RGB scene and a referring expression provided by a human using natural language, we aim to find the bounding boxes that show the described objects. To achieve this, we first use Grad-CAM (Selvaraju et al., 2017) to find the active areas in the scene, and then we use unsupervised clustering to find different clusters in these active areas. From these active clusters, we generate the bounding boxes most likely to belong to the target object regions (Figure 2).

#### 3.1.1 Obtaining heatmap activations

We use the image captioning module of Grad-CAM (Selvaraju et al., 2017) to find active areas of a scene. The module takes a scene and an expression as an input, and it generates a heatmap H as an output. This heatmap shows the relevant regions in the scene. In order to obtain the heatmap, the module uses the pre-trained NeuralTalk2 image captioning model (Karpathy and Fei-Fei, 2015) and finds the gradient of the caption’s log probability with respect to the final convolutional layer. Then, the module uses these gradients to provide visual explanations.

When different captions are provided for the same image, different areas become active depending on the items in the captions (e.g., different objects). In our work, these captions correspond to referring expressions, and we find the active areas specified by the referring expression (Figure 2A).

**FIGURE 3:**
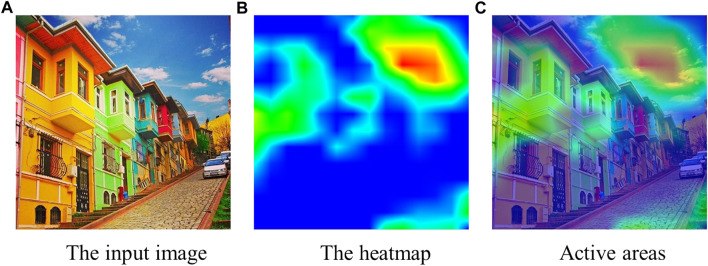
The input image (in **(A)**), the heatmap from Grad-CAM (in **(B)**), and the activations aligned with the original image (in **(C)**) when the expression is “the blue sky.” The Grad-CAM heatmap highlights the sky using the color features, although the sky is not in the object categories of the MSCOCO dataset.

Using the NeuralTalk2 image captioning model with Grad-CAM has the advantage of not being restricted to specific object categories. We achieve this because the NeuralTalk2 method was trained on a dataset (i.e., MSCOCO (Lin et al., 2014) with five captions per image collected from crowd workers) that describes scenes with many different features, not restricted to object categories. Thanks to varied scene descriptions encountered during the training of NeuralTalk2, when an object category is unknown (i.e., not in MSCOCO object categories), the higher-level feature space learned by NeuralTalk2 and visualized by Grad-CAM can be used to show the active areas that fit the given description. For instance, in Figure 3, when the expression is ‘the blue sky’, the highlighted region of Grad-CAM shows the sky, although the sky is not in the object categories of the MSCOCO. In that case, the color information is helpful for NeuralTalk2 to determine what to search for in the image. In this example, the existing works that first detect the candidate objects and select the target object among these candidates fail if they do not detect the sky, which is typically not recognized by off-the-shelf object detectors. On the other hand, by using the Grad-CAM activations of the NeuralTalk2 captioning method, we can consider the sky as a candidate region using the additional features given in the object description.

#### 3.1.2 Clustering heatmap

After finding the active areas in a scene, we aim to cluster them. These clusters can be interpreted as different regions belonging to candidate objects so the robot can direct its attention to the right part of the scene. To achieve this, we first find the total number of active areas in the heatmap and use this value to determine the number of the resulting active clusters. Consequently, we use K-means clustering to identify those clusters.

##### 3.1.2.1 Finding the number of clusters

In order to determine the number of clusters in K-means clustering, we find the number of unconnected areas that are active in the heatmap H . We first define a set U where its values are 1 for active pixels and 0 otherwise:

$$U=\left\{|p_{r}>T_{h}\;or\;p_{g}>T_{h}|,\;\forall p\in H\right\},$$ (1)

where |.| sets the value as 1 when the condition is correct and 0 otherwise. Additionally, *p*_*r*_ and *p*_*g*_ show the normalized intensity values of each pixel *p* for the red and green channels. We set the threshold *T*_*h*_ as 0.9 to only consider the pixels with high activation. A smaller value of this threshold can drastically increase the number of clusters by considering low-activation areas. With our formulation, U corresponds to all active areas in the heatmap. The visualization of U can be seen in Figure 2B.

After finding all active areas U , we compute the number of unconnected ones to determine the number of clusters. To this end, we consider the 2D connectivity of pixels. Concretely, two pixels are considered neighbors if they have horizontal, vertical, or diagonal connectivity, and their activations are the same (i.e., either 0 or 1). While computing the number of unconnected areas, we discard an area if it is very small (experimentally set as less than 150 pixels), and we consider the background to be another region. The calculated number of unconnected regions, *n*, is provided as the number of clusters for the K-means clustering algorithm.

##### 3.1.2.2 Using K-means clustering

**FIGURE 4:**
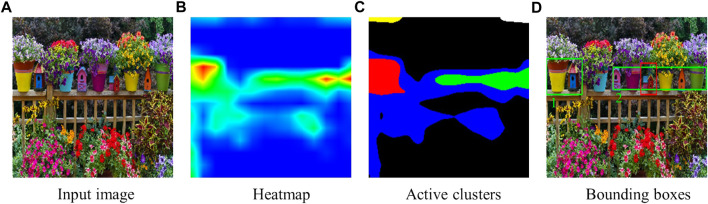
When the expression is “the red house between the pink and yellow flower pots,” **(A)** the input image, **(B)** the heatmap from Grad-CAM, **(C)** the active clusters from K-means, and **(D)** the suggested candidate bounding boxes (the green ones are the candidates and the red one is the target object). The activations of different regions overlap, so the heatmap is not straightforward to cluster without using K-means clustering.

For some activations in heatmaps, it can be challenging to determine whether close active areas belong to the same cluster. In these cases, the neighboring method explained in Section 3.1.2.1 can not be directly applied to separate the active regions into different clusters. For instance, in Figure 4, it is not possible to determine which active area belongs to which cluster by only checking their connectivity, given the activations of different regions overlap. To address this problem, we use K-means clustering.

In order to cluster each pixel *p*, we consider the following features: *f*(*p*) = {*p*_*x*_, *p*_*y*_, *p*_*r*_, *p*_*g*_, *p*_*b*_}. In our formulation, *p*_*x*_ and *p*_*y*_ are the normalized horizontal and vertical coordinates of pixel *p*. *p*_*r*_, *p*_*g*_ and *p*_*b*_ represent the normalized intensity values of the red, green and blue channels.

First, we apply a Gaussian filter to the heatmap H to smooth the image. The Gaussian kernel’s width and height are set as 11, and the smoothed image is represented as $H_{g}$ .

We define another set W such that every element in W corresponds to a pixel *p* and contains *f*(*p*) if *p* is active or zeros if *p* is inactive:

$$W=\left\{\|p_{r}>T_{m}\;or\;p_{g}>T_{m}\|,\;\forall p\in H_{g}\right\},$$ (2)

where ‖.‖ sets the value as *f*(*p*) when the condition is correct, and 0s otherwise. We set threshold *T*_*m*_ as 0.5 because we do not need to consider regions with low activation.

After finding the number of clusters, *n*, and features for each pixel in W , we cluster W using the K-means algorithm. The centroids of the clusters are initialized randomly, and they are updated by minimizing the within-cluster sum-of-squares. The maximum number of iterations for the algorithm is set to 300.

After obtaining the clusters from the K-means algorithm, we check whether there are unconnected regions within the same cluster. If a cluster has unconnected regions, we separate these regions into different clusters using 2D neighboring connectivity, as described in Section 3.1.2.1. Also, we discard a cluster if it is too small ( < 150 pixels). Therefore, the total number of clusters can be different than the *n* value.

We represent all of the obtained clusters as *C* and each cluster in *C* as *c*_*i*_–see Figure 2C for visualization of *C*. We calculate the activation of each cluster *c*_*i*_ ∈ *C* using the channel intensities in H :

$$a_{c_{i}}\leftarrow \frac{1}{n_{c_{i}}}\underset{\forall p\in c_{i}}{\sum }\left(w_{r}\times p_{r}+w_{g}\times p_{g}\right),\;for\;c_{i}\in C,$$ (3)

where *p*_*r*_ and *p*_*g*_ are the normalized red and green channel intensities in H , and $n_{c_{i}}$ represents the number of pixels in region *c*_*i*_. Further, *w*_*r*_ and *w*_*g*_ are the activation weights for the red and green channels. We experimentally set *w*_*r*_ as 0.7 and *w*_*g*_ as 0.3. *w*_*r*_ has a higher weight than *w*_*g*_ because red channels reflect more about the activation in our heatmap.

After finding activation value $a_{c_{i}}$ for each *c*_*i*_, we sort the clusters in descending order of their activation levels. We represent these sorted clusters as *C*_*sorted*_. For each *c*_*i*_ ∈ *C*_*sorted*_, we obtain the smallest bounding boxes covering *c*_*i*_. The obtained bounding boxes are represented as *B*_*sorted*_, and we consider *B*_*sorted*_ as the candidate bounding boxes most likely to belong to the described object.

The overall procedure of the Grad-CAM RGB method is summarized in Algorithm 1 (see Supplementary Material for an alternative solution).

Algorithm 1 Grad-CAM RGB method.

**Input:** an RGB scene and a referring expression.
**Output:** *B*_*sorted*_, the candidate bounding boxes belonging to the described object.
1 Generate the heatmap H using Grad-CAM for the given scene and the referring expression
2 Set U to sbe the all active areas in H (Eq. 1)
3 Let *n* to be the number of disconnected areas in U

4 Obtain $H_{g}$ by applying a Gaussian filter to H

5 Let W to contain the feature vectors of pixels in $H_{g}$ (Eq. 2)
6 Cluster W using K-means clustering with *n* number of clusters
7 Set *C* to be the clusters obtained from K-means clustering
8 Calculate the activation $a_{c_{i}}$ for each cluster *c*_*i*_ ∈ *C* (Eq. 3)
9 Obtain *C*_*sorted*_ by sorting *C* in terms of the cluster activations
10 Set *B*_*sorted*_ to be the smallest bounding boxes covering each cluster in *C*_*sorted*_
11 Provide *B*_*sorted*_ as the candidate bounding boxes belonging to the described object

### 3.2 Grad-CAM RGB-D method

**FIGURE 5:**
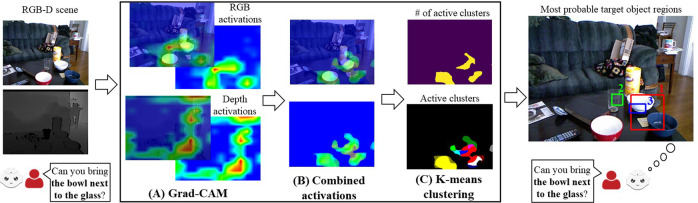
For a given RGB-D scene and a referring expression (i.e., the bold part of the user expression), the overview of the Grad-CAM RGB-D method to obtain the bounding boxes containing the target object regions. The RGB and depth heatmaps generated by Grad-CAM in **(A)**, combined activations in **(B)**, and the results from K-means clustering in **(C)**.

To obtain the described RGB-D scene regions for a given expression, we propose to extend the Grad-CAM RGB method presented in Section 3.1 with depth features. To achieve this, we first generate the activation heatmap of RGB and depth channels using Grad-CAM. Then, we find the combined activations showing the common active areas in these channels. Finally, we apply K-means clustering to the combined activations and suggest the bounding boxes covering the clusters with the highest activations as the regions belonging to the described objects. (See Figure 5 for an overview.)

#### 3.2.1 Obtaining heatmap activations

**FIGURE 6:**
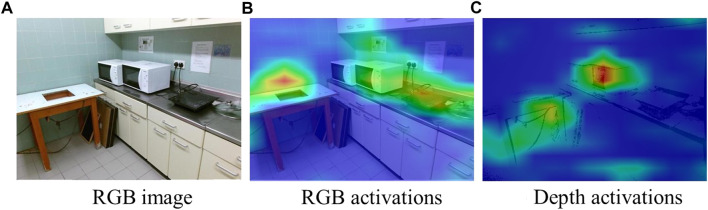
The RGB image (in **(A)**), the heatmap activations of RGB image (in **(B)**), and depth activations (in **(C)**) when the expression is “the microwave closer to the table.”

To obtain the active parts of RGB-D scenes, we use the NeuralTalk2 image captioning module of Grad-CAM as in Section 3.1.1. The NeuralTalk2 image captioning model was trained on RGB images, but thanks to its rich feature space, the Grad-CAM activations of the captioning model can also generate useful activations for the depth dimension of the scenes. For instance, in Figure 6, heatmap activations of NeuralTalk2 in RGB image are not accurate enough to identify ‘the microwave closer to the table’. On the other hand, the heatmap of the depth image forces these activations towards the described area. Therefore, in this case, using the depth heatmap together with the RGB one can help to highlight the correct areas.

After observing the depth heatmap can help to identify the areas described by a user, as in Figure 6, we provide an RGB-D scene to Grad-CAM through its RGB channels and depth dimension. Therefore, we obtain two different heatmaps, one from RGB denoted as $H^{\mathrm{RGB}}$ and another from the depth denoted as $H^{\mathrm{depth}}$ . For instance, in Figure 5A, the image in the back in the first row shows $H^{\mathrm{RGB}}$ and the image in the back in the second row visualizes the $H^{\mathrm{depth}}$ .

In the heatmap representation, higher intensities in the red channel show higher activations, and higher values in the blue channels denote lower heatmap activations as before. We represent each pixel’s normalized RGB channel intensities as $\left\{p_{r}^{\mathrm{RGB}},p_{g}^{\mathrm{RGB}},p_{b}^{\mathrm{RGB}}\right\}$ and $\left\{p_{r}^{\mathrm{depth}},p_{g}^{\mathrm{depth}},p_{b}^{\mathrm{depth}}\right\}$ for $H^{\mathrm{RGB}}$ and $H^{\mathrm{depth}}$ respectively.

#### 3.2.2 Combining RBG and depth activations

After obtaining the activation heatmaps $H^{\mathrm{RGB}}$ and $H^{\mathrm{depth}}$ , we find the intersecting area of the active parts in the heatmaps. First, we check the channel intensities of each pixel for both $H^{\mathrm{RGB}}$ and $H^{\mathrm{depth}}$ . When red or green channel intensities are higher than a threshold *T*_*rgb*_ (experimentally set as 0.39) for both of the pixels in $H^{\mathrm{RGB}}$ and $H^{\mathrm{depth}}$ , we assume that the corresponding pixel in their intersection heatmap $H^{\mathrm{int}}$ is also active. In that case, we take the mean of each channel in $H^{\mathrm{RGB}}$ and $H^{\mathrm{depth}}$ to set the corresponding pixel intensities $\left\{p_{r}^{\mathrm{int}},p_{g}^{\mathrm{int}},p_{b}^{\mathrm{int}}\right\}$ in $H^{\mathrm{int}}$ :

$$p_{r}^{\mathrm{int}}\leftarrow \frac{1}{2}\left(p_{r}^{\mathrm{RGB}}+p_{r}^{\mathrm{depth}}\right),$$ (4)

$$p_{g}^{\mathrm{int}}\leftarrow \frac{1}{2}\left(p_{g}^{\mathrm{RGB}}+p_{g}^{\mathrm{depth}}\right),$$ (5)

$$p_{b}^{\mathrm{int}}\leftarrow \frac{1}{2}\left(p_{b}^{\mathrm{RGB}}+p_{b}^{\mathrm{depth}}\right).$$ (6)

If the red and green channels of a pixel in $H^{\mathrm{RGB}}$ or $H^{\mathrm{depth}}$ are lower than *T*_*rgb*_, we set the corresponding pixel in $H^{\mathrm{int}}$ as inactive, i.e., we set $\left\{p_{r}^{\mathrm{int}},p_{g}^{\mathrm{int}},p_{b}^{\mathrm{int}}\right\}$ as {0, 0, 1} since the highest intensity in blue channel shows an inactive pixel. The second row of Figure 5B shows an example visualization of $H^{\mathrm{int}}$ .

#### 3.2.3 Clustering heatmap

After obtaining $H^{\mathrm{int}}$ showing the activation intersection of $H^{\mathrm{RGB}}$ and $H^{\mathrm{depth}}$ , we cluster $H^{\mathrm{int}}$ to find the active regions in the RGB-D scene. To achieve this, we first obtain the number of clusters and then use this number for K-means clustering to identify the active clusters.

To obtain the number of clusters *n* from $H^{\mathrm{int}}$ , we calculated the number of unconnected areas in $H^{\mathrm{int}}$ following the procedure explained in Section 3.1.2.1 – see the first row of Figure 5C for the visualization of number of active clusters. The computed number *n* is provided as the number of clusters to the K-means clustering.

After finding the cluster count *n*, we apply K-Means clustering to determine the active clusters. We first apply a Gaussian filter to $H^{\mathrm{int}}$ as in Section 3.1.2.2 and obtained $H_{g}^{\mathrm{int}}$ . Then, we define a feature vector for each pixel in $H_{g}^{\mathrm{int}}$ . After the Gaussian smoothing, if a pixel is active (i.e., the red or blue channel has a value higher than 0.5, as before), the feature vector of the pixel contains the five features descried in Section3.1.2.2 and also the depth feature:

$$f\left(p^{\mathrm{int}}\right)=\left\{p_{x}^{\mathrm{int}},p_{y}^{\mathrm{int}},p_{z}^{\mathrm{int}},p_{r}^{\mathrm{int}},p_{g}^{\mathrm{int}},p_{b}^{\mathrm{int}}\right\},$$ (7)

where these features correspond to the pixel’s coordinates in the *x* and *y*-axes, its corresponding depth value obtained from the input RGB-D scene, and its pixel intensities in red, blue, and green channels, respectively. All of these feature values are normalized in the zero to one range. Alternatively, if a pixel is not active after smoothing, the feature vector is set as {0, 0, 0, 0, 0, 0}, as before.

Using the pixels’ features and the calculated number of clusters *n*, we cluster the pixels of $H_{g}^{\mathrm{int}}$ with K-means clustering following the procedure explained in Section 3.1.2.2—see the second row of Figure 5C for the visualization of example clusters. After the K-means clustering, we calculate the activation $a_{c_{i}}$ of each cluster *c*_*i*_ using the Eq. 3, and sort the clusters from the highest activation to the lowest. Finally, we suggest the bounding boxes *B*_*sorted*_ covering the sorted clusters as the candidate bounding boxes containing the target object.

Algorithm 2 summarizes the overall procedure of the Grad-CAM RGB-D method.

Algorithm 2 Grad-CAM RGB-D Method.

**Input:** an RGB-D scene and a referring expression.
**Output:** *B*_*sorted*_, the candidate bounding boxes belonging to the described object.
1 Generate the heatmap activations $H^{\mathrm{RGB}}$ and $H^{\mathrm{depth}}$ using Grad-CAM
2 Find the heatmap $H^{\mathrm{int}}$ showing the common active areas of $H^{\mathrm{RGB}}$ and $H^{\mathrm{depth}}$ (Eqs 4, 5, 6)
3 Count the number of unconnected areas (*n*) of active pixels in $H^{\mathrm{int}}$

4 Obtain $H_{g}^{\mathrm{int}}$ by applying a Gaussian filter to $H^{\mathrm{int}}$

5 Collect the feature vector of each pixel in $H_{g}^{\mathrm{int}}$ (Eq. 7)
6 Find the clusters by employing K-means clustering to the feature vector with *n* number of clusters
7 Follow the steps between the lines 7–10 in Algorithm 1, and obtain *B*_*sorted*_
8 Suggest *B*_*sorted*_ as candidate bounding boxes showing the target object regions

## 4 Experiments and results

To evaluate the Grad-CAM RGB and RGB-D methods, we conducted two sets of experiments. First, to assess the Grad-CAM RGB method efficacy, we selected a state-of-the-art referring expression comprehension method as a baseline (i.e., MAttNet (Yu et al., 2018)), gathered varied real-world images, and compared the results of both methods on these images. Then, to analyze the impacts of depth features, we compared the Grad-CAM RGB-D method with the Grad-CAM RGB method on another dataset containing depth dependent and independent features.

### 4.1 MattNet baseline

For a given RGB scene and referring expression, MAttNet first obtains the candidate objects using an object detection module. Then, the method checks how well the expression fits each of the candidate objects. Finally, the candidate object that best fits the expression is considered the target object. To compare the Grad-CAM RGB method with MAttNet, we sort the candidate bounding boxes by how well they fit the expression. Similar to Grad-CAM RGB output, the bounding boxes ordered from the most likely to the least likely are considered MAttNet’s candidate bounding boxes belonging to the described object.

### 4.2 Data collection

#### 4.2.1 MTurk dataset

To compare the Grad-CAM RGB method with MAttNet, we gathered a dataset of 25 images containing indoor and outdoor scenes (12 images from SUN (Xiao et al., 2010), 8 images from Google Images, 4 images from Doğan et al. (2019), and 1 image from SUN RGB-D (Song et al., 2015)). These images are classified as easy (7 images), medium (8 images), and hard (10 images) difficulty levels. An image is labeled as easy if there are only a few objects in total, they are commonly known objects (e.g., bottle, book, mouse, *etc.*), and the number of same-type objects is 2 (i.e., only one distractor per object). If the objects are common, but the number of distractors is at least three per object, the image is classified in the medium category. The images in the hard group contain many objects with distractors and some objects that are not so common (e.g., radish, papaya, and artichoke). Since MAttNet uses Mask R-CNN (He et al., 2017) for extracting objects, we determine an object as common if it is part of the list of instance categories of Mask R-CNN (i.e., 90 types of objects), so a fair comparison is ensured. Next, one target object per image is annotated by a person blind to our research questions (female, 29 years old). She was instructed to draw a bounding box around an object she would consider difficult to describe.

Thereafter, we used Amazon Mechanical Turk (AMT) to collect written expressions describing the target objects in the images. We asked AMT workers to provide an unambiguous description of the target object such that it could be differentiated from other similar objects in the image and gave them some examples. We asked them to describe the objects to a robot in order to collect descriptions that simulate interactions between a user and a robot (e.g., a user requests an object from a robot). For each interaction, each user could describe an object using its various features or refer to an object in relation to other objects. For example, different AMT workers described the object in Figure 8D as ‘the brown vegetable on the top right’, ‘the purple vegetable right next to the mushrooms’, and ‘the turnip to the right of the eggplant’. To account for this variability, we gathered 10 expressions describing the same target object in the same image. In total, we obtained 250 expressions–see Figure 8 for some examples.

We gathered such a dataset to evaluate the Grad-CAM RGB method’s performance in different conditions. The easy and medium difficulty images represent the typical computer vision datasets for referring expression comprehension (e.g., RefCOCO dataset (Yu et al., 2016) which contains MSCOCO (Lin et al., 2014) images where MAttNet and NeuralTalk2 were trained). In these scenes, the total object categories are limited (91 novel object categories for COCO images) and detectable by existing object detectors. On the other hand, in the hard category dataset, the object categories go beyond the existing datasets, and this dataset represents the scenes that can be encountered in the wild. Therefore, this three-level difficulty dataset enables us to observe the behavior of the methods in many interactions at different difficulty levels. Further, neither NeuralTalk2 nor MAttNet were trained on our collected scenes and expressions, which helps us to better evaluate the methods’ generalization capacities.

#### 4.2.2 SUN RGB-D dataset

To compare the Grad-CAM RGB and RGB-D methods, we gathered another dataset with 70 scenes from SUN RGB-D (Song et al., 2015). This dataset contains various real-world scenes collected from different spatial contexts (e.g., living room, bedroom, bathroom, office, *etc.*). Moreover, for each scene, we selected a target object with at least one distractor (i.e., the objects that are in the same object category as the target object). Further, for each target object, we collected an expression describing the target object in a natural and unambiguous manner. In the end, we obtained a dataset with 70 images and 70 expressions referring to the target objects.

Half of our dataset (35 images) was considered to be depth independent, and the remaining half was labeled as depth dependent. In depth independent category, the target objects were described with features that were not tied to depth dimensions (e.g., the spatial relations such as ‘to the left’, ‘to the right’ or other object features such as the color or object type. In contrast, the depth dependent category images needed the depth dimension to disambiguate the target objects. Therefore, the expressions used to describe the target objects were dependent on their three-dimensional distances (e.g., the expressions contained depth-dependent spatial relations such as ‘close by’, ‘next to’, ‘in front of’, *etc.*) – see Figure 10 for some example images and expressions).

We collected such a dataset because we aim to assess the impacts of using depth features for dept dependent and independent environments. The instances that we collected for this purpose enable us to manipulate the environment’s depth dependence for a detailed comparison of the Grad-CAM RGB and RGB-D methods. Moreover, the equal proportion of instances for each category ensures the fair evaluation of the methods’ overall performance.

### 4.3 Evaluation procedure

The candidate bounding boxes are obtained from the MTurk dataset using Grad-CAM RGB and MattNet, and from the SUN RGB-D dataset using the Grad-CAM RGB and RGB-D methods. The first three candidates from each method are considered for computing a matching score with the target object bounding box. To calculate the matching score, *S*_*i*_, we use 1 − *L*_*DIoU*_, where *L*_*DIoU*_ (defined by Zheng et al. (2020)) represents the matching loss function between two bounding boxes. Therefore, *S*_*i*_ is:

$$S_{i}\leftarrow \frac{area\left(b_{i}\cap b_{\mathrm{target}}\right)}{area\left(b_{i}\cup b_{\mathrm{target}}\right)}-\frac{d^{2}}{c^{2}},$$ (8)

where *b*_*i*_ is the candidate bounding box and *b*_*target*_ is the box of the target object. *d* represents the normalized distance between the centers of *b*_*i*_ and *b*_*target*_, and c is the normalized diagonal length of the smallest box covering *b*_*i*_ and *b*_*target*_.

In Eq. 8, the first term gives a higher score for a higher intersection of the boxes, and the second term penalizes the distance between their center of masses. The matching score *S*_*i*_ can vary in [−1,1] interval. The first of the three candidates that results in *S*_*i*_ > 0 is accepted as the candidate box showing the region belonging to the target object. In the case of all three candidates having a score lower than zero, we report it as none of the candidate boxes belonging to the target object.

In the first evaluation with the MTurk dataset, the same steps were applied to the Grad-CAM RGB method and MatNett for the 250 expressions. Both methods could find at least three candidate boxes in all cases, except for MAttNet in one instance. That image belongs to the easy category, and it was able to find the target object for the first two candidates without affecting the reported results.

In the second evaluation with the SUN RGB-D dataset, the same steps were applied to the Grad-CAM RGB and RGB-D methods for 70 expressions, and both of the methods could suggest at least three candidate boxes.

### 4.4 Results

#### 4.4.1 Grad-CAM RGB vs. MattNet baseline

**FIGURE 7:**
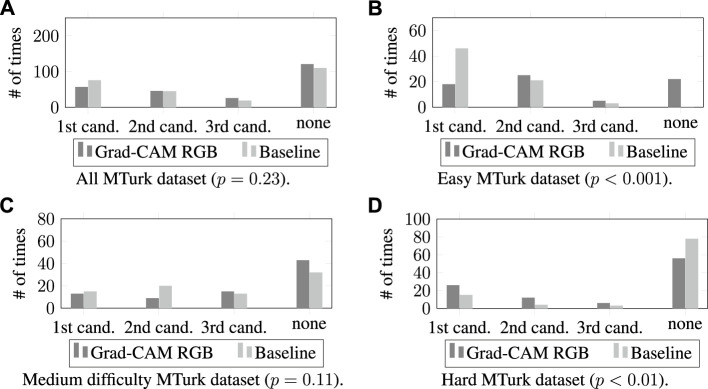
The number of times the Grad-CAM RGB method and the Mattnet baseline generated candidate bounding boxes that matched the target object by difficulty level. All MTurk Dataset in **(A)**, easy images in **(B)**, medium difficulty images in **(C)**, and hard images in **(D)**.

**FIGURE 8:**
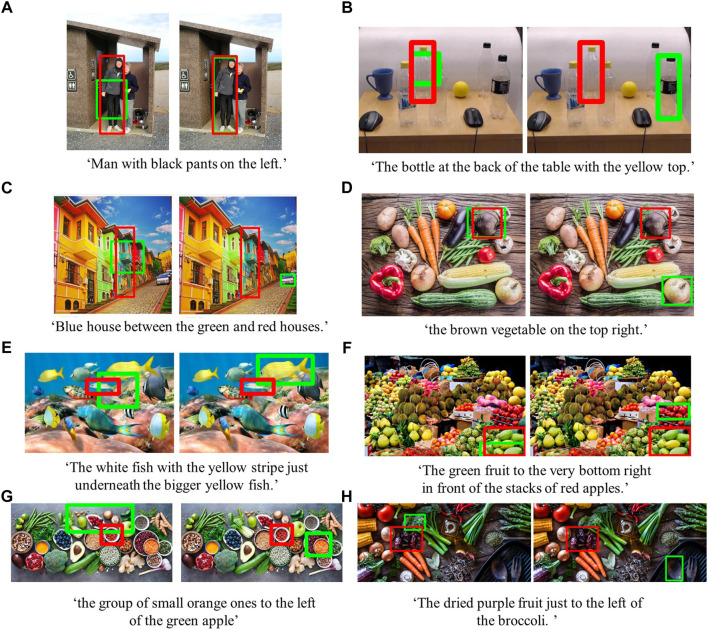
Examples of easy **(A)**, medium **(B)**, and hard **(C,D,E,F,G, H)** MTurk dataset images with original expressions collected from AMT workers describing the target objects in red boxes. The green boxes indicate the first proposed candidate object from the Grad-CAM RGB method (on the left) and the MAttNet baseline (on the right). Best viewed in color.

In this section, we present our results comparing the Grad-CAM RGB method with the MattNet baseline in the MTurk dataset for 250 expressions. Figure 7 presents how often the target object matched with the first three candidates for all images at each level of difficulty. In Figure 8, we show the first candidates suggested by the two methods for the same images and target objects.

##### 4.4.1.1 All MTurk dataset

We first compared the Grad-CAM RGB method with MAttNet for how many times the target object from the 250 user expressions matched the first, second, or third candidate bounding boxes according to the *S*_*i*_ score from Eq 8. A Chi-Square test did not find any significant differences between the methods, *χ* ^2^ (3, *N* = 500) = 4.34, *p* = .23. Most often, the target object was not matched with any of the first three candidate bounding boxes proposed by the two models (i.e., the mode was “none” of the candidates for both methods). In Figure 7A, we can see that both methods showed similar trends for different candidates, and the number of times that the methods generated candidate bounding boxes that matched the target object were similar.

##### 4.4.1.2 Easy MTurk dataset

We examined the results for the easy images with 70 expressions (Figure 7B). We conducted a two-sided Fisher’s exact test (the minimum expected value was less than 5 for some cells, so the Chi-Square test couldn’t be applied). The results showed significant differences (Fisher’s exact test value: 40.29, *N* = 140, *p* < 0.001. Most often, the target object was matched with the first candidate bounding box for the MAttNet baseline and second candidate for the Grad-CAM RGB method–see Figure 7B). Examining the first candidate, the baseline found the target objects more often than the Grad-CAM RGB method did. Moreover, there were no cases where none of the baseline’s first three candidates was correct, while the Grad-CAM RGB method had 22 cases.

##### 4.4.1.3 Medium difficulty MTurk dataset

For the medium difficulty images, we evaluated the results for 80 expressions. A Chi-Square test did not identify a significant difference between the methods (*χ* ^2^ (3, *N* = 160) = 6.07, *p* = .11, the mode was “none” of the candidates for both methods). Figure 7C shows that the number of times finding the target boxes was similar for the first and third candidates for both methods. The results from both methods were slightly different for the second and the last items, but these differences were not significant.

##### 4.4.1.4 Hard MTurk dataset

We compared the Grad-CAM RGB method with the baseline for the hard category scenes for 100 expressions (Figure 7D). We again conducted a two-sided Fisher’s exact test, that showed significant differences (Fisher’s exact test value: 11.44, *N* = 200, *p* = .009, the mode was “none” of the candidates for both methods). The results indicate that the Grad-CAM RGB method found the target object in its first, second, and third candidates more often than MAttNet. Also, the baseline had a higher number of cases for which no candidate was correct.

#### 4.4.2 Grad-CAM RGB-D vs. Grad-CAM RGB

**FIGURE 9:**
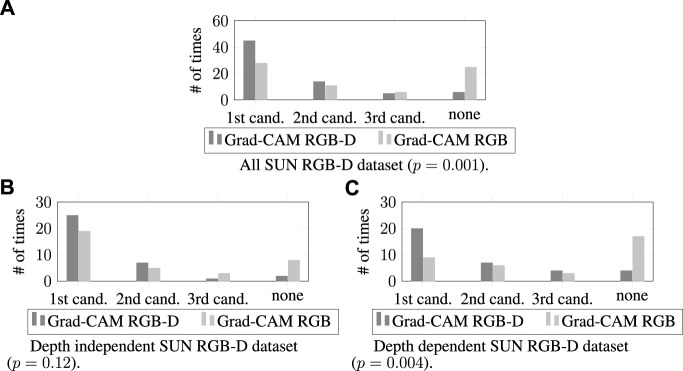
The number of times that the generated candidate bounding boxes matched with the target objects for the all SUN RGB-D dataset (in **(A)**), depth independent (in **(B)**), and dependent (in **(C)**) categories.

**FIGURE 10:**
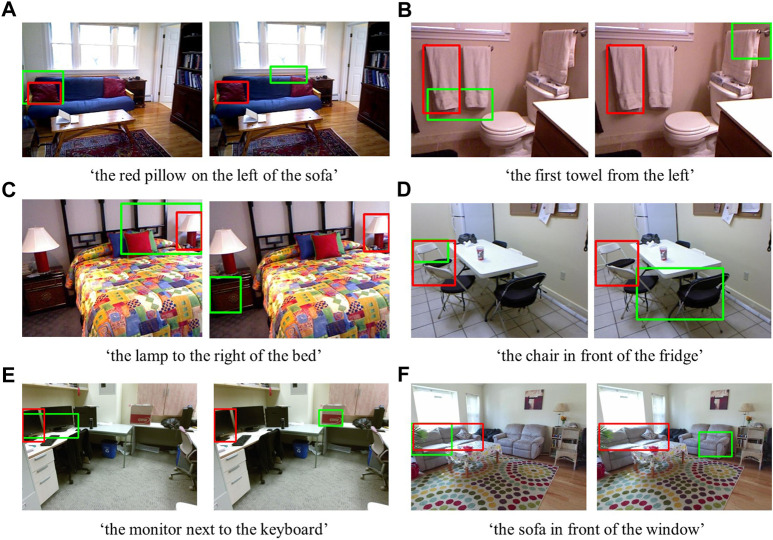
Examples from the depth independent **(A,B, C)** and depth dependent **(D,E, F)** SUN RGB-D dataset. The red bounding boxes show the target objects (ground truth), and the green boxes show the first candidates from the Grad-CAM RGB-D method (on the left) and the Grad-CAM RGB method (on the right) suggested for the given expressions. Best viewed in color.

We compared the Grad-CAM RGB-D method with the Grad-CAM RGB method considering the number of times the target object matched with the candidate bounding boxes in the SUN RGB-D dataset for different depth dependencies–see Figure 9. Further, we provided some qualitative examples showing the first candidate bounding boxes suggested by both methods for the depth independent and dependent categories (Figure 10).

##### 4.4.2.1 All SUN RGB-D dataset

We first evaluated our results by considering the whole SUN RGB-D dataset (70 images). Figure 9A shows that the Grad-CAM RGB-D method found the target object more often in its first and second candidates compared to the Grad-CAM RGB method. Moreover, the cases where none of the first three candidates matched with the target object were rarer in the Grad-CAM RGB-D method. Further analysis of these results with a Chi-Squared test showed that these differences were significant (*χ* ^2^ (3, *N* = 140) = 16.06, *p* = .001; the mode is the first candidate for both methods, i.e., the candidate most often matched with the target object was the first candidate).

##### 4.4.2.2 Depth independent SUN RGB-D dataset

To assess the impacts of depth features, we also examined the results in the depth independent category (35 images), where the target object descriptions did not depend on depth. Figure 9B shows that the Grad-CAM RGB-D method’s first and second candidates matched with the target object more often, and the Grad-CAM RGB-D method failed less while suggesting the regions belonging to the target object. However, when we examined the results with Fisher’s exact test (a Chi-Squared test could not be applied because some cells had a minimum expected value of fewer than five), we did not observe any significant differences between methods (Fisher’s exact test value: 5.59, *N* = 70, *p* = 0.12, the mode is the first candidate for both methods).

##### 4.4.2.3 Depth dependent SUN RGB-D dataset

Finally, we evaluated the impacts of using the depth dimension for the depth dependent category (35 images), where the descriptions of the target objects’ were tied to their depth features. The results shown in Figure 9C demonstrated that the regions identified by the Grad-CAM RGB-D method in its first, second, or third candidates matched with the target object more often compared to the RGB method. Further, the Grad-CAM RGB-D method had fewer cases where none of its first three candidates matched the target object. To assess these results’ significance, we ran another Fisher’s exact test. The result of this analysis showed that the differences were significant (Fisher’s exact test value: 12.67, *N* = 70, *p* = 0.004; the mode is the first candidate for the Grad-CAM RGB-D method and none of the first three candidates for the Grad-CAM RGB method).

## 5 Discussion

In this section, we discuss our results where we compared the Grad-CAM RGB method with the MattNet baseline on the MTurk dataset, and also the analysis obtained from the evaluation of the Grad-CAM RGB and RGB-D methods on the SUN-RGB-D dataset.

First of all, MAttNet performs significantly better than the Grad-CAM RGB method for easy MTurk images. This was expected because there are few objects in the images, the number of distractors per object is only one, and the objects are commonly known. Therefore, the chance level for MAttNet to predict the target is very high (i.e., 1/*n* where *n* is the total number of detected objects). The chance level is lower for the Grad-CAM RGB method because it focuses on the activation of each pixel, not the detected object boxes.

The results for hard MTurk images show that the Grad-CAM RGB method performs significantly better than the MAttNet baseline at suggesting regions belonging to the target object. This shows that the Grad-CAM RGB method can be employed when MattNet fails to identify target objects in challenging environments where there are many objects with distractors and also uncommon objects. In these environments, the users mostly referred to the uncommon objects using features such as color, shape, general category (e.g., vegetable instead of radish), and their spatial relationships with known objects nearby. On the other hand, in the easy and medium difficulty MTurk images, the users described the objects primarily using the objects’ exact names because they are familiar. Therefore, the results indicate that the Grad-CAM RGB method performs better than MAttNet when the descriptions are based on an object’s features instead of its name.

We did not expect to observe significant differences for the all MTurk dataset and medium difficulty MTurk images because our goal with the Grad-CAM RGB method is not an overall performance improvement, given that it does not simplify the problem to select the target object among the suggested candidates. Instead, we aim to suggest a method that can work better *in the wild* (e.g., with uncommon objects and ambiguities). Therefore, the hard MTurk dataset is crucial for the evaluation of such a system. Results on this dataset are critical for human-robot collaboration because it is impossible to assume that the robot is familiar with all of the different ways that users will use when referring to objects in the real world. In these cases, the Grad-CAM RGB method successfully suggests regions by using known concepts. For instance, in Figure 8D, if the robot doesn’t know the concept of a vegetable, it can still predict a region by looking for something brown and on the top right. In other words, the Grad-CAM RGB method can handle the unknown objects in the expressions by employing explainability of image captioning and looking for which input features (i.e., which pixels of the image in our case) contribute more to the output. However, handling unknown objects is more difficult for the MAttNet baseline because there should be a detected bounding box to consider an object as a candidate.

From the qualitative results of the MTurk dataset, we observe that the Grad-CAM RGB method focuses on the regions which are important for the given expression. For instance, in Figure 8A from the easy MTurk images, the Grad-CAM RGB method finds a bounding box focused on the pants of the man because the expression includes this information. From the same example, we also notice that the bounding box suggested by the Grad-CAM RGB method does not entirely cover the man, but MAttNet provides more precise bounding boxes in such cases (commonly known objects with fewer ambiguities) by being based on an object detector. On the other hand, when there are uncommon objects (e.g., papayas in Figure 8F), relying on important regions of the scene, not only specified by object categories but also object features, enables the Grad-CAM RGB method to find regions that better fit expressions than MAttNet. Even in the failure cases shown in Figures 8G,H (reported as none in Figure 7), the suggested regions are still sensible. For instance, in Figure 8H, the suggested bounding box focuses on the broccoli because the expression includes this information. This is crucial because our goal with the Grad-CAM RGB method is to endow robots with the ability to direct their attention to the right part of the scene in the wild and determine the regions to ask for an efficient follow-up clarification instead of asking the user to repeat the whole request again.

In line with our goal, our qualitative results from the MTurk dataset support that if there are ambiguities in the environment, the Grad-CAM RGB method can be used to ask for further clarifications by only focusing on the active clusters instead of the whole image. For example, when we asked AMT workers to describe objects as if describing them to a robot (i.e., to obtain object descriptions simulating natural language user requests), there were ambiguities in their descriptions. For instance, in Figure 8E, the worker’s description fits both of the small white fishes, and the bounding box obtained from the Grad-CAM RGB method contains the parts of both fishes. In another example, when the description is the green vegetables in Figure 2, the Grad-CAM RGB method finds the active clusters on the green vegetables for the first two candidates. Also, in Figure 4, when the red birdhouse is described, the Grad-CAM RGB method finds the most active regions on the birdhouses. Therefore, these examples demonstrate that the robot can ask the user to clarify the request by only considering these active regions instead of taking into account the whole images (e.g. in Figure 8E, the robot can ask ‘do you mean the fish on the left or on the right?’). In brief, focusing on active clusters can improve the efficiency of human-robot collaboration.

When we compared the Grad-CAM RGB and RGB-D methods to see whether using depth features improves the system performance, the quantitative evaluation for depth independent SUN RGB-D dataset demonstrated that using the depth of the objects did not result in significant differences. In this category, similar performances from the Grad-CAM RGB and RGB-D methods were expected because the target object descriptions are not dependent on the depth dimension. However, the system performance was significantly improved for the whole SUN RGB-D dataset and the depth dependent category. Further, the improvement was even more distinct for the depth dependent instances. The performance advancements in this category, which was collected to simulate depth-dependent environments, show that considering depth is critical in real-world applications of referring expression comprehension. In these applications, the objects are located in three-dimensional feature space, and finding the described object can be impossible without their depth features. In such cases, when the robot is comprehending the user’s expressions, the Grad-CAM RGB-D method can be used for successful human-robot collaboration.

Our quantitative results from the all SUN RGB-D dataset and depth dependent category also demonstrated that the Grad-CAM RGB-D method could identify the target objects in its first candidate more often than the Grad-CAM RGB method could. Furthermore, the number of failures (i.e., none of the first three candidates matched with the target object) was significantly fewer for the Grad-CAM RGB-D method in these cases. These findings imply that, in a real-world environment, the robot would find the described objects more often in its first selection without opting for its latter candidates, and it would make fewer mistakes if the depth dimension were provided in its input space. This suggests that using depth while comprehending users’ expressions improves the task accuracy and efficiency of human-robot collaboration.

In our qualitative results from the depth independent SUN RGB-D category, we show the first candidate bounding boxes suggested by the Grad-CAM RGB-D and RGB methods in Figures 10A,B,C. Even though we did not observe significant differences in our quantitative results for this category, the qualitative results show some of the examples in which the RGB-D method (on the left) suggested the regions matching the described objects better than the RGB method (on the right). Although some bounding boxes from the Grad-CAM RGB-D method do not exactly cover the target objects, the suggested regions are still sensible. For instance, the region suggested in Figure 10C partially contains the lamp and the bed when the expression is ‘the lamp to the right of the bed’. However, the region suggested by the Grad-CAM RGB method is towards the incorrect lamp. Therefore, significant differences between methods for this category might be obtained with further analysis of the suggested regions by using different matching scores or asking users to evaluate these proposed regions.

In our qualitative results for the depth dependent SUN RGB-D category (Figures 10D,E,F), we show the first candidate bounding boxes obtained from the Grad-CAM RGB-D (on the left) and RGB methods (on the right). We observe that the regions suggested by the Grad-CAM RGB-D method fit better to the target object. In these examples, the lack of depth features misleads the Grad-CAM RGB method to select the distractor objects. For example, in Figure 10D, when the expression is ‘the chair in front of the fridge,’ the Grad-CAM RGB method highlighted the incorrect chairs, which can be considered in front of the fridge in 2D. However, the Grad-CAM RGB-D method can handle these situations using the additional features obtained from the depth dimension. These examples demonstrate the significance of the depth features for accurate comprehension of referring expressions in real-world environments.

## 6 Conclusion and future work

We propose the Grad-CAM RGB method to point the robot’s attention in the regions of a scene described by a user to improve human-robot collaboration in the wild and also suggest extending this method to Grad-CAM RGB-D considering the depth features. Our methods find the regions belonging to the described objects using explainability. In the Grad-CAM RGB method, the region activations of an RGB scene are found using Grad-CAM, and then we use K-means clustering to obtain the active clusters. On the other hand, the Grad-CAM RGB-D method uses Grad-CAM to generate the activation heatmaps of RGB channels and the depth dimension, and then the combined activations, obtained from the common active parts of the heatmaps, are clustered to find the active clusters showing the target object. Our qualitative results from the Grad-CAM RGB method demonstrate that the regions suggested by this method can be used to resolve ambiguities. Moreover, through our evaluation, we show that the Grad-CAM RGB method works better than a state-of-art baseline for scenes with uncommon objects and multiple distractors. Finally, we demonstrate that using the depth dimension in the Grad-CAM RGB-D method significantly improves the performance in depth dependent and the whole evaluation dataset, which includes all of the depth dependent and independent category instances.

There could be several extensions of our work. We have already deployed the Grad-CAM RGB method in a robot to evaluate the efficiency of the interaction while resolving the ambiguities by asking follow-up clarifications (Doğan et al., 2022). This interaction can be further examined with the perspective of explainable robotics (Setchi et al., 2020) considering how users’ perception of the robot is affected by the given visual explanations of the system predictions. Additionally, although we use the NeuralTalk2 image captaining model to obtain the activation heatmaps, our approach is applicable to other CNN-based image captioning models, such as Huang et al. (2019) and Jiang et al. (2018). Therefore, future research can make use of our method and utilize other state-of-art captioning techniques to possibly improve the presented accuracies. Further, our system can be expanded by taking into account the aspects of visual attention studies (e.g., the importance of surrounding context (Itti and Koch, 2001) or correlation between the visual attention and gaze (Borji and Itti, 2013; Zaraki et al., 2014)). Moreover, the Grad-CAM module can be used to take the three dimensions (i.e., an RGB-D scene) as an input instead of obtaining RGB and depth activations separately. In this case, the challenge can be training an image captioning network that performs well in 3D scenes to visualize the RGB-D gradient activations. Although there are recent attempts to address the image captioning task in 3D (e.g., Chen et al. (2021)), these studies focus on relatively small datasets compared to MSCOCO, and the varied scene descriptions of MSCOCO enable our approach to work for uncommon object categories. If RGB-D gradient activations can be obtained from such a rich dataset, our method can be applied to them to obtain the described object regions without putting any restrictions on object categories. Finally, 3D point clouds can be provided in the input space instead of RGB-D images, and the performance of the robot can be evaluated further with and without depth features. This interaction can also be examined for the user’s trust and reliance on the system predictions, which are critical measures for explainable robotics.

## Data Availability

The raw data supporting the conclusion of this article will be made available by the authors, without undue reservation.
