# Supplementary material for: Leveraging explainability for understanding object descriptions in ambiguous 3D environments
Source: Front Robot AI. 2023 Jan 4;9:937772. doi: 10.3389/frobt.2022.937772 (PMC9872646; doi:10.3389/frobt.2022.937772)
Supplement: Supplementary file 1 [file DataSheet1.pdf]

Supplementary Material

1 ALTERNATIVE SOLUTION: USING ACTIVATION MAPS INSTEAD OF HEATMAPS

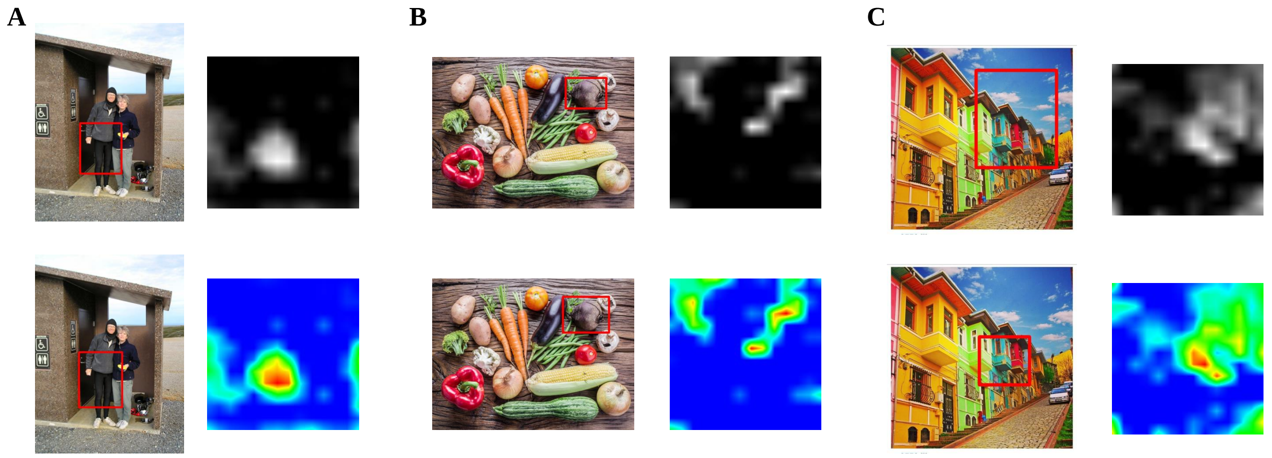

"Man with black pants on the left." "the brown vegetable on the top right." "Blue house between the green and red houses."

Figure S1. Bounding boxes obtained by using activation maps (first row) and heatmaps (second row) for an MTurk dataset easy images image in (**A**) and hard images in (**B**) and (**C**).

As an alternative solution to the Grad-CAM RGB method, instead of obtaining active regions from a heatmap $\mathcal{H}$, these regions can be obtained from an activation map $\mathcal{A}$, where $\mathcal{A}$ is the probability map obtained from Grad-CAM, and $\mathcal{H}$ is RGB-mapping of these activations. In this alternative solution, $\mathcal{A}$ can be obtained from the Grad-CAM method as before using the captaining module. As shown in Figure S1, the activation map $\mathcal{A}$ is a grayscale image (the first row), and the heatmap $\mathcal{H}$ is its corresponding color-mapped RGB image (the second row).

After obtaining $\mathcal{A}$ using Grad-CAM, Algorithm 1 can be followed as before. The main difference is that instead of five features for each pixel p, there are three features when $\mathcal{A}$ is used: $f(p) = \{p_x,\ p_y,\ p_a\}$. In this notation, p_x and p_y are the normalized horizontal and vertical coordinates of pixel p, and p_a is the normalized activation of the pixel obtained from $\mathcal{A}$. Accordingly, $\mathcal{U}$ (in Equation 1), $\mathcal{W}$ (in Equation 2) and a_{c_i} (in Equation 3) can be computed as follows:

$$\mathcal{U} = \{|p_a > T_h\,|, \quad \forall p \in \mathcal{A}\}, \tag{S1}$$

$$\mathcal{W} = \{||p_a > T_m\,||, \quad \forall p \in \mathcal{A}_g\}, \tag{S2}$$

$$a_{c_i} \leftarrow \frac{1}{n_{c_i}} \sum_{\forall p \in c_i} p_a, \quad \text{for } c_i \in C, \tag{S3}$$

where $\mathcal{A}_g$ represents after applying a Gaussian filter to $\mathcal{A}$. Finally, B_{sorted} can be obtained as before.

When we compare the bounding boxes suggested by this alternative approach to the ones obtained from heatmaps (Figure S1), we observe slight differences in the areas covered by the boxes, but the focal points do not change in either approach under the assumption that the described object should be located close

to the center of bounding boxes, given these are the most active regions. On the other hand, heatmaps bring the advantage of providing a more transparent and explainable solution by better visualizing the areas contributing to the predictions. Also, heatmaps offer more importance to the activations with three features (p_r, p_g, and p_b) compared to a single one (p_a). Therefore we propose the Grad-CAM RGB method by utilizing RGB heatmaps instead of grayscale activation maps in the paper manuscript.
